
JOURNAL INFORMATION
==============================
NLM Title Abbreviation: Wirtschaftsdienst
Journal ID: Wirtschaftsdienst
NLM Journal ID: 101086612

Wirtschaftsdienst (Hamburg, Germany : 1949)

ISSN: 0043-6275

ARTICLE INFORMATION
==============================
PMCID: PMC7174811
PMID: 32336795
DOI: 10.1007/s10273-020-2617-3
Article ID: 2617
Article version: 1
Subjects: Leitartikel

Corona-Krise trifft auf Strukturprobleme im Gesundheitswesen

The corona crisis meets the structural healthcare problems

Schreyögg Jonas jonas.schreyoegg@wiso.uni-hamburg.de

LS für Management im Gesundheitswesen, Hamburg Center for Health Economics, Esplanade 36, 20354 Hamburg, Deutschland
Electronic publication date: 2020 Apr 22
Print publication date: 2020
Volume: 100
Issue: 4
First page: 226
Last page: 227
Copyright: © Der/die Autor(en) 2020
License: Open Access Dieser Artikel wird unter der Creative Commons Namensnennung 4.0 International Lizenz veröffentlicht, welche die Nutzung, Vervielfältigung, Bearbeitung, Verbreitung und Wiedergabe in jeglichem Medium und Format erlaubt, sofern Sie den/die ursprünglichen Autor(en) und die Quelle ordnungsgemäß nennen, einen Link zur Creative Commons Lizenz beifügen und angeben, ob Änderungen vorgenommen wurden.
Die in diesem Artikel enthaltenen Bilder und sonstiges Drittmaterial unterliegen ebenfalls der genannten Creative Commons Lizenz, sofern sich aus der Abbildungslegende nichts anderes ergibt. Sofern das betreffende Material nicht unter der genannten Creative Commons Lizenz steht und die betreffende Handlung nicht nach gesetzlichen Vorschriften erlaubt ist, ist für die oben aufgeführten Weiterverwendungen des Materials die Einwilligung des jeweiligen Rechteinhabers einzuholen.
Weitere Details zur Lizenz entnehmen Sie bitte der Lizenzinformation auf http://creativecommons.org/licenses/by/4.0/deed.de.
License URL: https://creativecommons.org/licenses/by/4.0/

issue-copyright-statement: © The Author(s) 2020

==============================
Jonas Schreyögg ist Inhaber des Lehrstuhls für Management im Gesundheitswesen und wissenschaftlicher Direktor am Hamburg Center for Health Economics sowie Mitglied des Sachverständigenrats Gesundheit.

Die Corona-Krise stellt viele gesellschaftliche Bereiche vor besondere Herausforderungen. Langjährige strukturelle Probleme treten nun auf besondere Weise zutage. Im Bereich der digitalen Infrastruktur betrifft dies den fehlenden Breitbandausbau, der die Produktivität ganzer Landstriche im Homeoffice gefährdet. Auch die strukturellen Probleme des deutschen Gesundheitswesens werden in dieser Krise besonders sichtbar und müssen nun kreativ quasi über Nacht zumindest provisorisch gelöst werden. Einige dieser Probleme seien im Folgenden dargestellt.

Bereits bei Verdacht auf SARS-CoV-2 offenbart sich die erste Problematik des deutschen Gesundheitssystems. In den letzten Jahren ist ein deutlicher Trend erkennbar, dass Patienten mit Symptomen jeglicher Art direkt die Notaufnahme eines Krankenhauses aufsuchen. Seit einiger Zeit versuchen zwar die Kassenärztlichen Vereinigungen, die Patienten dazu zu bewegen, außerhalb der Sprechstundenzeiten, den bei nicht lebensbedrohlichen Zuständen zuständigen ärztlichen Bereitschaftsdienst unter der Telefonnummer 116 117 zu kontaktieren. Bis dato schien der Weg direkt in die Krankenhäuser allerdings mehr den Präferenzen der Bürger zu entsprechen. Dies führte am Anfang der Pandemie zu gefährlichen Situationen in Notaufnahmen von Krankenhäusern. Ein neues Gesetz zur Notfallversorgung, das sich gerade im Gesetzgebungsprozess befindet, sollte hier Abhilfe schaffen und die fragmentierte Notfallversorgung in Deutschland insgesamt besser organisieren. Nun musste die Gesundheitspolitik jedoch durch massive öffentliche Kommunikation die Patientenströme schnell in Richtung der telefonischen Ersteinschätzung über die 116 117 umsteuern.

Das nächste Strukturproblem zeigt sich bei der Nachverfolgung von Kontaktfällen sowie der Meldung von Infizierten, für die der öffentliche Gesundheitsdienst (ÖGD) in den kommunalen Gesundheitsämtern zuständig ist. In einem Gesundheitssystem, das sich in den letzten Jahrzehnten zunehmend auf Kuration ausgerichtet hat, wurde der für Prävention, Gesundheitsförderung und Gesundheitsschutz zuständige ÖGD stetig personell reduziert. Entsprechend ist der ÖGD kapazitär nicht auf die aktuellen Anforderungen eingestellt. In vielen Kommunen wurde nun durch Medizinstudierende und Verwaltungsmitarbeitende aus anderen Bereichen zumindest kurzfristig Entlastung geschaffen.

Bei der Meldung der Infizierten durch die Kommunen und Landesbehörden, aber auch der Krankenhäuser an das Robert Koch-Institut (RKI) zeigt sich dann im nächsten Schritt das gesamte Digitalisierungsdilemma des deutschen Gesundheitswesens. So werden unter anderem Zahlen gefaxt, per Telefon gemeldet, händisch in Computer eingegeben. Es ist daher derzeit durch Zeitverzögerungen und Unschärfen schwierig, sich ein genaues Bild des Ausbruchsgeschehens und der Versorgungslage zu machen. Dabei war die Gesundheitspolitik gerade auf dem Weg, die Digitalisierung im Gesundheitswesen voranzubringen. Auch wenn die Notlage hier einen gewissen Digitalisierungsschub bewirkt hat, kann sie natürlich nicht das gesamte Gesundheitswesen über Nacht digitalisieren.

Im Mittelpunkt der Krise stehen die Krankenhäuser. Es ist hinlänglich bekannt, dass Deutschland im internationalen Vergleich sehr viele Fälle stationär versorgt (insbesondere bei Operationen), die andere Länder gar nicht oder ambulant behandeln. Dadurch haben wir nicht nur viele nicht bedarfsnotwendige Krankenhäuser und Betten, sondern vor allem das Problem, dass das vorhandene pflegerische und medizinische Personal auf zu viele Krankenhäuser verteilt wird. Genau diese Problematik tritt nun zutage, denn eine hohe Zahl an aufgestellten Krankenhausbetten heißt noch nicht, dass ein Gesundheitssystem für die Behandlung von COVID-19-Patienten besonders gut gerüstet wäre. Vielmehr sind da sehr spezielle Kenntnisse in Infektiologie auf internistischen Stationen sowie für die Beatmung auf Intensivstationen erforderlich. Diese Kenntnisse sind vor allem bei Universitätskliniken und Maximalversorgern, d. h. in der Regel sehr großen Krankenhäusern mit sehr differenzierter medizinisch-technischer und personeller Ausstattung, vorhanden. Der Begriff Krankenhaus der Maximalversorgung ist nicht eindeutig definiert. Zählt man die Krankenhäuser ab 500 Betten so umfasst dies ca. 271 Krankenhäuser in Deutschland. Viele dieser Maximalversorger haben zu wenige IntensivpflegerInnen, um die sehr personalintensiven COVID-19-Patienten zu behandeln, denn das vorhandene Personal teilt sich eben in Deutschland auf viele Kliniken auf, darunter Kliniken, die nicht für die Behandlung von COVID-19-Patienten ausgestattet sind.

Hinzu kommt das Problem, dass die Maximalversorger in der deutschen Krankenhausvergütung in den letzten Jahren zunehmend unter die Räder geraten sind. Das System der diagnosebezogenen Fallgruppen (DRG-System) mit Fallpauschalen verfolgt den sogenannten Ein-Haus-Ansatz, d. h. differenziert nicht nach Versorgungsstufen. Entsprechend werden Maximalversorger und Universitätskliniken im Prinzip wie jedes andere Krankenhaus vergütet, obwohl sie andere Vorhaltekosten haben, unter anderem komplexe technische Ausstattungen für hochkomplexe Fälle, die per definitionem gar nicht voll ausgelastet werden können. Da zusätzlich die Investitionszuschüsse der Länder in den letzten Jahrzehnten deutlich reduziert wurden, sind die entsprechenden Krankenhäuser aber auf Überschüsse dringend angewiesen. Als Folge wurde vielerorts die Pflegepersonaldecke ausgedünnt, obwohl der Zusammenhang zwischen der Personalbesetzung mit gut ausgebildeten PflegerInnen und der Versorgungsqualität (unter anderem die Mortalität) in vielen Studien für verschiedene Krankheitsbilder gezeigt wurde. Auch hier war der Gesetzgeber gerade mit Pflegeuntergrenzen auf dem Wege, eine Besserung herbeizuführen. Die Effekte konnten sich aber noch nicht entfalten.

Angesichts dieser Probleme ist die intendierte Verdopplung der Intensivkapazitäten eine gewaltige Aufgabe, die durch kreative Lösungen vorangebracht wird. So wurde das Personal vielerorts durch Freiwillige, Rückkehrer aus anderen Jobs und Medizinstudierende aufgestockt, um zumindest die ÄrztInnen und IntensivpflegerInnen von anderen Tätigkeiten zu entlasten. Vor dem Hintergrund dieser Strukturprobleme muss man konstatieren, dass es der deutschen Gesundheitspolitik gut gelungen ist, das Gesundheitswesen kurzfristig für die Corona-Krise zu rüsten. Insgesamt hat die Krise das deutsche Gesundheitswesen zu einer ungünstigen Zeit getroffen, da die Gesundheitspolitik gerade dabei war, viele überfällige Strukturreformen anzugehen. Zu diesen zählen eine Reform der Notfallversorgung, eine Reform der sektorübergreifenden Vergütung und vor allem die Implementierung der Digitalisierung im Gesundheitswesen. Darüber hinaus wären eine Krankenhausstrukturreform und -vergütungsreform notwendig. In fünf bis zehn Jahren hätte uns diese Krise vermutlich mit geeigneteren Strukturen getroffen und weniger Rüstkosten für eine kurzfristige Vorbereitung erfordert. Die aktuelle Krise zeigt deshalb sehr klar, wie wichtig ein konsequentes Vorantreiben von Strukturreformen ist.

Jonas Schreyögg
Universität Hamburg
jonas.schreyoegg@uni-hamburg.de
